# Refining the Moderate Inclusion Range of Dried Asian Watermeal (*Wolffia globosa*) in the Diets of Two-Spotted Crickets (*Gryllus bimaculatus*): Integrating Segmented Regression and Nutritional Self-Selection

**DOI:** 10.3390/insects17040420

**Published:** 2026-04-15

**Authors:** Jamlong Mitchaothai, Rachakris Lertpatarakomol, Achara Lukkananukool, Tassanee Trairatapiwan, Natnaree Kaewsiri, Nils T. Grabowski

**Affiliations:** 1 Office of Administrative Interdisciplinary Program on Agricultural Technology, School of Agricultural Technology, King Mongkut’s Institute of Technology, Ladkrabang (KMITL), Bangkok 10520, Thailand; jamlong.mi@kmitl.ac.th; 2 Faculty of Veterinary Medicine, Mahanakorn University of Technology (MUT), Bangkok 10530, Thailand; rachakris@mut.ac.th (R.L.); tassanee@mut.ac.th (T.T.); natnaree@mut.ac.th (N.K.); 3 Department of Animal Production Technology and Fisheries, School of Agricultural Technology, King Mongkut’s Institute of Technology, Ladkrabang (KMITL), Bangkok 10520, Thailand; 4 Institute for Food Quality and Food Safety, University of Veterinary Medicine Hannover (TiHo), 30173 Hannover, Germany; nils.grabowski@tiho-hannover.de

**Keywords:** *Wolffia globosa*, *Gryllus bimaculatus*, nutritional geometry, segmented regression, nutrient partitioning, insect nutrition

## Abstract

The integration of rapidly renewable biomass into insect production systems has been proposed as a strategy to improve resource-use efficiency in insect production. This study evaluated the graded inclusion levels of dried watermeal (*Wolffia globosa*) in diets of two-spotted crickets (*Gryllus bimaculatus*) and assessed voluntary nutrient regulation under free-choice feeding. Four fixed-inclusion diets (0%, 25%, 35%, and 45% watermeal) and one self-selection treatment were tested over 28 days. Growth performance, feed conversion ratio (FCR), survival rate (Surv), production index (PI), and whole-body composition were determined. Repeated-measures analysis using linear mixed-effects models indicated that treatment, week, and their interaction were statistically significant (*p* ≤ 0.024). However, partial R^2^ analysis showed that the independent contributions of treatment and week were negligible, whereas the treatment × week interaction explained measurable variance, indicating that dietary effects were primarily expressed through time-dependent responses. Segmented regression identified a breakpoint at 35% watermeal inclusion (95% CI: 24.93–45.07), indicating that PI was the highest within a moderate supplementation range under the present fixed-diet conditions rather than at a precise single optimum. Inclusion levels beyond this threshold reduced performance. Under free-choice conditions, crickets progressively increased watermeal intake with age and maintained stable nitrogen-free extract (NFE):crude protein (CP) and gross energy (GE):CP intake ratios, selecting an average of 25–35% watermeal over the experimental period. This supplementation range improved feed efficiency and protein deposition while limiting lipid accumulation, suggesting improved energy–protein balance and nutrient partitioning. The self-selection result is interpreted as evidence of behavioral intake regulation under choice conditions and not as direct validation of the segmented-regression breakpoint. Collectively, these findings provide complementary statistical and behavioral evidence supporting a biologically relevant moderate inclusion range (approximately 30–35%) of dried watermeal.

## Simple Summary

Sustainable feed ingredients are essential for improving the environmental performance of insect farming. This study evaluated dried watermeal (*Wolffia globosa*), a fast-growing aquatic plant, as a partial replacement for commercial feed in two-spotted crickets (*Gryllus bimaculatus*). Crickets were fed diets containing 0%, 25%, 35%, or 45% watermeal, and an additional group was allowed to choose freely between commercial feed and watermeal. Growth performance, feed efficiency, survival, and body composition were measured over four weeks. The results showed that moderate inclusion levels improved growth and feed conversion, while excessive inclusion reduced performance. Segmented regression suggested that, under the present fixed-diet conditions, the highest production index occurred within a moderate inclusion range centered near 35% watermeal, although the estimated breakpoint was associated with a broad confidence interval. When given a dietary choice, crickets voluntarily selected approximately 25–35% watermeal over time, indicating behavioral intake regulation under choice conditions, rather than direct validation of a single optimal level. This supplementation range increased protein deposition and reduced fat accumulation, indicating improved nutrient use efficiency. These findings suggest that moderate inclusion of dried watermeal may be suitable for the formulated diets of two-spotted crickets, though further studies are needed to refine practical inclusion recommendations.

## 1. Introduction

The transition toward a bio-circular and green economy requires food production systems that maximize nutrient-use efficiency while minimizing environmental burden [1,2]. Conventional livestock production is associated with high land demand, water consumption, and greenhouse gas emissions [3,4]. In contrast, insect farming has emerged as a resource-efficient alternative, capable of converting plant-derived substrates into high-quality protein with substantially lower ecological footprints [5,6]. Integrating insect production with locally renewable biomass and nutrient recycling systems represents a promising pathway toward circular protein supply chains [7,8,9].

Cricket farming, particularly of the two-spotted cricket (*Gryllus bimaculatus*), has expanded rapidly in Asia [10] and is increasingly recognized as a scalable source of alternative protein [11]. However, the sustainability of cricket production depends strongly on feed formulation. Many commercial feeds rely on soybean meal and other globally traded protein sources linked to land-use change and competition with human food systems [12,13,14]. Substituting these ingredients with rapidly renewable, regionally cultivable biomass could improve both environmental and economic resilience. From a life cycle assessment (LCA) perspective, feed production is consistently identified as the dominant contributor to the environmental footprint of insect farming systems, accounting for a substantial proportion of greenhouse gas emissions, land use, and energy demand. Previous LCA studies of insect production systems have demonstrated that the environmental performance of insects relative to conventional livestock is highly dependent on the type and origin of feed substrates, with feed production and composition being major determinants of greenhouse gas emissions, land use, and overall environmental footprint [15,16]. For example, the use of conventional feed ingredients such as soybean meal or cereal-based diets can significantly increase environmental burdens, whereas the incorporation of low-impact or residual biomass streams can markedly reduce the overall footprint of insect production systems. Therefore, optimizing feed formulation is not only a matter of improving biological performance but also a critical lever for mitigating environmental impacts and enhancing the sustainability of insect-based protein production [16,17].

Watermeal (*Wolffia globosa*), one of the smallest flowering aquatic plants, represents a promising circular biomass candidate [18,19,20]. It exhibits rapid growth, high crude protein (CP) content, and efficient nutrient uptake, particularly nitrogen and phosphorus [18,21], and is also rich in vitamin B_12_ [22]. Watermeal can be cultivated in compact aquatic systems and potentially integrated into nutrient recovery loops [20], such as aquaculture effluents or wastewater-derived nutrient streams. Conceptually, such a system forms a closed nutrient loop: residual nutrients → watermeal biomass production → insect feed → cricket biomass → frass return to crop production or watermeal cultivation [23]. This circular nutrient-loop model reduces external feed inputs and enhances system-level sustainability.

Beyond sustainability considerations, dietary watermeal supplementation may influence cricket performance through changes in nutrient balance that may have metabolic implications. In insects, carbohydrates derived from nitrogen-free extract (NFE) serve as primary substrates for glycolysis and subsequent adenosine triphosphate (ATP) production via the tricarboxylic acid (TCA) cycle [24,25,26]. Adequate ATP availability is essential for peptide bond formation, amino acid incorporation, and protein accretion. When carbohydrate supply exceeds anabolic demand, excess acetyl-CoA may be redirected toward de novo lipogenesis in the fat body, promoting lipid storage. Conversely, insufficient non-protein energy may result in amino acid oxidation to meet energetic demands, reducing net protein deposition. Therefore, the dietary energy-to-protein balance (e.g., gross energy (GE):CP ratio) has been suggested to play an important role in nutrient partitioning between lean tissue growth and lipid accumulation [27,28,29]. These concepts provide a physiological framework for interpretation, but the present study did not directly measure these pathways.

In a recent study, Mitchaothai et al. [19] evaluated graded levels (0–100%) of dried watermeal supplementation in commercial diets for *G. bimaculatus* and reported that performance was maintained with up to 50% inclusion, while higher levels reduced feed intake and survival [19]. Using segmented regression analysis, a breakpoint associated with the highest PI based on a composite PI was estimated at 36.7% [19]. That nonlinear response suggested that moderate watermeal supplementation may be associated with improved overall production performance, although the underlying physiological mechanisms were not assessed directly. However, the supplementation intervals tested (25% and 50%) left a relatively broad range around the predicted breakpoint unexplored. While the previous study [19] established a general dose–response relationship and identified a broad optimal inclusion level (≈36.7%) using segmented regression, the present study extends this work by refining the dose–response resolution around the predicted breakpoint (25%, 35%, and 45%), integrating a self-selection feeding approach to assess behavioral nutrient regulation, and examining nutrient intake patterns (e.g., GE:CP and NFE:CP ratios) to provide mechanistic insight into nutrient partitioning. Collectively, these advances provide a more refined and biologically informed basis for evaluating moderate inclusion ranges in cricket feed formulation.

Insects are also capable of active macronutrient regulation, as described by the nutritional geometry framework [30,31,32]. Under free-choice feeding conditions, orthopterans can adjust intake proportions to approach an intake target that maximizes growth efficiency and metabolic balance [33]. Because insects are capable of active macronutrient regulation under free-choice feeding conditions, crickets provided with dietary choices may voluntarily approach an intake proportion that is biologically informative in relation to performance responses observed under fixed-diet conditions. However, the self-selection treatment represents a distinct feeding architecture and was therefore used to evaluate behavioral intake regulation rather than to directly validate the regression-derived breakpoint from the fixed-inclusion treatments. Accordingly, the objectives of the present study were to (i) refine the evaluation of dried watermeal supplementation by testing inclusion levels centered around the previously predicted breakpoint (0%, 25%, 35%, and 45%), and (ii) assess voluntary dietary selection when crickets were provided separate access to commercial feed and dried watermeal. The authors hypothesized that (1) production performance would follow a nonlinear dose–response relationship with a breakpoint near 35% watermeal inclusion, potentially consistent with differences in nutrient balance, and (2) under self-selection conditions, crickets would voluntarily approach a watermeal intake proportion reflecting behavioral nutrient regulation. By integrating segmented regression analysis with behavioral observations under self-selection, this study aims to provide complementary statistical and biological context for evaluating watermeal supplementation in a circular cricket production system.

## 2. Materials and Methods

### 2.1. Experimental Site and Rearing Conditions

The experiment was conducted at the insect production facility of the School of Agricultural Technology, King Mongkut’s Institute of Technology, Ladkrabang (KMITL), Bangkok, Thailand. All procedures were conducted in accordance with institutional guidelines for animal care and use, as described in the “Institutional Review Board Statement” section.

Eggs of the two-spotted cricket (*G. bimaculatus*) were obtained from our in-house breeding colony maintained under an ambient rearing system. After hatching, newly emerged nymphs (<24 h old) were randomly allocated to experimental units. A total of 120 nymphs were stocked per plastic rearing box (24.7 × 35.7 × 32.3 cm; width × length × height), with six replicates per treatment. Each box was equipped with horizontally arranged egg cartons to increase surface area and reduce crowding stress. Water was supplied daily using moistened gauze placed in shallow trays. Boxes were covered with nylon mesh to ensure ventilation and prevent contamination or predator access. Crickets were maintained under ambient tropical conditions throughout the 28-day experimental period, with room temperature maintained at approximately 29–30 °C, and relative humidity (approximately 60%), under a 12 h light:12 h dark photoperiod. Environmental conditions were monitored routinely throughout the study to confirm consistency across the rearing period. Frass and debris were removed weekly. Management practices were standardized across all treatments. Detailed descriptions of the rearing and management procedures are provided in a previous publication [19].

### 2.2. Experimental Diets, Watermeal Preparation, and Feeding Design

Commercial feed (Betagro 260^®^, Betagro Public Company Limited, Lop Buri, Thailand) contained 22.94% CP, 3.35% crude fat, and 3917.52 kcal/kg of GE (Table 1). Two feed components were used: the abovementioned commercial cricket feed (fine powder form) and dried Asian watermeal (*W. globosa*). The dried Asian watermeal was obtained from a commercial supplier in Mahasarakham, Thailand. Fresh biomass was harvested from cultivated water ponds and sun-dried to a constant dry matter content. The dried material was ground into a fine powder, stored in sealed plastic bags at 4 °C in a dark environment, and used within 2 months for diet formulation. Prior to use, samples were visually inspected for contamination and subjected to proximate analysis. No visible spoilage, mold contamination, or abnormal odor was detected before use. Both ingredients were subjected to proximate analysis prior to diet formulation (Table 1). Because a single batch was used, formal batch-to-batch variation was not evaluated in this experiment. Drinking water was supplied daily using moistened, clean gauze placed in a small plastic tray (9 cm × 15 cm × 2.5 cm) with a rough surface.

#### 2.2.1. Fixed-Inclusion Treatments (T1–T4)

Four experimental diets were formulated by partially replacing the commercial feed with dried watermeal at inclusion levels of 0% (control; 100% commercial feed, T1), 25% (T2), 35% (T3), and 45% (T4) on an as-fed basis. Diets were mixed thoroughly to ensure uniformity and were provided ad libitum in low-profile plastic feeding trays (9 cm × 15 cm × 2.5 cm) with a rough surface.

#### 2.2.2. Self-Selection Treatment (T5)

In treatment T5, crickets were allowed to self-select between the two feed components. Commercial feed and dried watermeal were offered simultaneously in separate but identical feeding trays within each rearing box. Both feeds were provided ad libitum throughout the experimental period. Feed trays were positioned symmetrically and rotated periodically to minimize positional bias.

### 2.3. Feed Intake Determination and Voluntary Selection

The feed intake and body weight of the two studied two-spotted crickets were recorded weekly (days 7, 14, 21, and 28). For each rearing box, crickets were hand-counted and weighed using a digital balance (Sartorius^®^, Sartorius AG, Göttingen, Germany).

#### 2.3.1. Feed Intake of Crickets in Fixed-Inclusion Treatments (T1–T4)

Feed intake per replicate was calculated as the difference between feed offered and feed refused. Intake was expressed as total intake per replicate (g) and per cricket (mg/cricket).

#### 2.3.2. Feed Intake of Crickets in the Self-Selection Treatment (T5)

For T5, the intake of each feed component was determined separately as follows: Commercial feed intake = commercial feed offered − commercial feed refused; Watermeal intake = watermeal offered − watermeal refused. Total intake was calculated as: Total intake = commercial feed intake + watermeal intake. The voluntary proportion of watermeal in the selected diet was calculated as: Watermeal proportion (%) = (watermeal intake × 100)/total intake. Similarly, the proportion of commercial feed selected was calculated as: Commercial feed proportion (%) = (commercial feed intake × 100)/total intake. These values were used to quantify dietary preference under free-choice feeding conditions.

### 2.4. Growth Performance, Survival Rate, and PI

Crickets were weighed weekly by collectively weighing all individuals within each replicate using a calibrated digital balance. The following performance parameters were calculated: body weight (BW), body weight gain (BWG), average daily feed intake (ADFI), average daily gain (ADG), feed conversion ratio (FCR), survival rate (Surv), and PI.

Feed conversion ratio was calculated as:

$$FCR=\frac{\mathrm{Total}\;\mathrm{feed}\;\mathrm{intake}}{\mathrm{Total}\;\mathrm{weight}\;\mathrm{gain}}$$

Survival rate (%) was calculated weekly as:

$$\mathrm{Surv}=\frac{\mathrm{Number}\;\mathrm{of}\;\mathrm{live}\;\mathrm{crickets}}{\mathrm{Initial}\;\mathrm{number}}\times 100$$

To integrate performance variables, a PI was calculated as:

$$PI=\frac{\mathrm{ADG}\;(\mathrm{mg})\times \mathrm{Survival}\;\mathrm{rate}\;(\%)}{\mathrm{FCR}\times 10}$$

This index integrates growth rate, feed efficiency, and survival into a single performance indicator [19]. The PI is a composite indicator that simultaneously captures growth rate (ADG), feed utilization efficiency (FCR), and survivability. This integrative approach is conceptually aligned with production efficiency indices widely used in poultry and livestock systems (e.g., European Broiler Index; EBI, and European Production Efficiency Factor; EPEF), where optimal performance, farm management, and health are achieved [34,35] through the combined maximization of growth and survival while minimizing feed input. In the context of insect production, PI provides a holistic measure of system productivity by reflecting the balance between biomass gain, resource use efficiency, and population retention. Accordingly, PI was used as the primary response variable for segmented regression because the present study aimed to identify a practically relevant supplementation range for overall production performance under rearing conditions, rather than to optimize any single biological trait in isolation. Therefore, PI should be interpreted as an integrative production endpoint rather than a direct biological variable.

### 2.5. Sample Collection and Chemical Analysis

On day 28, crickets were fasted for 4 h to allow gut clearance and subsequently harvested. Equal numbers of male and female crickets were collected from each experimental unit and stored at −20 °C until analysis. Prior to analysis, samples were dried at 60 °C for 72 h and ground to a particle size of 0.5 mm. Proximate composition (dry matter, CP, crude fat, crude fiber; CF, and ash) was determined following standard AOAC procedures. CP of diet samples was calculated as nitrogen × 6.25. For cricket samples, nitrogen × 5.0 was used to account for non-protein nitrogen associated with chitin [11,36].

### 2.6. Statistical Analysis

All statistical procedures were conducted using R software (R Foundation for Statistical Computing, Vienna, Austria; version 4.5.1). The experimental unit for all analyses was the replicate rearing container (*n* = 6 per treatment), and data are presented as mean ± standard deviation (SD). Statistical significance was declared at *p* < 0.05. For segmented regression analysis, replicate-level data from the four fixed-inclusion treatments (T1–T4) were pooled, resulting in a total sample size of 24 experimental units. Repeated-measures analyses of production performance variables, including ADFI, BWG, ADG, FCR, Surv, and PI, were conducted using replicate-level observations across all five treatments (T1–T5; *n* = 30 experimental units), with treatment, week, and their interaction included in the model. Repeated-measures analysis of feed intake in the self-selection treatment (T5) was conducted separately to evaluate age-dependent intake patterns, using replicate as the experimental unit (*n* = 6), with week as the within-subject factor. Overall growth performance and whole-body composition were analyzed using one-way ANOVA based on replicate-level data (*n* = 6 per treatment).

#### 2.6.1. Repeated-Measures Analysis of Production Performance in Crickets Across Experimental Treatments (T1–T5)

Repeated-measures data for production performance variables, including ADFI, BWG, ADG, FCR, Surv, and PI, were analyzed using linear mixed-effects models (LMM) implemented in the lme4 package. The model was specified as:

$$Y_{ijk}=\mu +T_{i}+W_{j}+(T\times W{)}_{ij}+R_{k}+\varepsilon_{ijk}$$

where $Y_{ijk}$ is the observed response, μ  is the overall mean, $T_{i\;}$ is the fixed effect of treatment, $W_{j\;}$ is the fixed effect of week (age), ${\left(T\times W\right)}_{ij}$ is the interaction term, $R_{k\;}$ is the random effect of replicate, and $\varepsilon_{ijk\;}$ is the residual error. Significance of fixed effects was evaluated using Type III Analysis of Variance (ANOVA) with the lmerTest package. Estimated marginal means were obtained using emmeans, and pairwise comparisons among treatments within each week were conducted using Tukey-adjusted multiple comparisons. Compact letter displays were generated using the cld() function. Effect sizes were quantified using partial R^2^ with bootstrap confidence intervals (CIs) and calculated using the partR^2^ package to estimate the unique contribution of treatment, week, and their interaction after accounting for shared variance. Effect sizes are reported to complement significance testing and facilitate interpretation of biologically meaningful variance.

Diagnostic checks: Model assumptions were evaluated based on residual diagnostics. Normality of residuals was assessed using quantile–quantile (Q–Q) plots and the Shapiro–Wilk test. Homogeneity of variance was evaluated by visual inspection of residuals versus fitted values plots. Independence of residuals within each experimental unit was addressed through the inclusion of replicate as a random effect. Model fit and potential outliers were assessed using standardized residuals and influence diagnostics. When minor deviations from normality or homoscedasticity were observed, models were retained due to the robustness of LMM to such violations.

#### 2.6.2. Repeated-Measures Analysis of Feed Intake in Crickets Under T5

For the self-selection treatment (T5), repeated-measures analysis of feed intake variables (commercial feed intake, watermeal intake, total intake, and watermeal proportion) was conducted separately to evaluate the effect of week (age). A linear mixed-effects model was applied with week as a fixed effect and replicate as a random effect:

$$Y_{jk}=\mu +W_{j}+R_{k}+\varepsilon_{jk}$$

where $Y_{jk}\;$ is the observed intake variable, $W_{j}\;$ is the fixed effect of week, $R_{k\;}$ is the random effect of replicate, and $\varepsilon_{jk\;}$ is the residual error. Pairwise comparisons among weeks were performed using Tukey-adjusted multiple comparisons based on estimated marginal means. Week 1 watermeal intake was excluded from inferential analysis due to zero intake across replicates. This absence of intake likely reflects early developmental or behavioral factors (e.g., initial feed acceptance, palatability, particle size, or novelty of the ingredient), rather than solely nutrient regulation.

Diagnostic checks: Residual diagnostics were conducted as described above, including Q–Q plots, residual versus fitted plots, and assessment of standardized residuals. Model assumptions were considered acceptable based on visual inspection and the robustness of mixed-effects models.

#### 2.6.3. Overall Performance and Body Composition

Overall 4-week performance data and whole-body nutrient composition were analyzed using one-way ANOVA:

$$Y_{i}=\mu +T_{i}+\varepsilon_{i}$$

where $T_{i\;}$ represents the fixed effect of treatment. Models were fitted using aov() or lm(), and treatment means were compared using Tukey’s test.

Diagnostic checks: ANOVA assumptions were evaluated using Shapiro–Wilk tests for normality and Levene’s test for homogeneity of variance. Residual plots were also visually inspected. When assumptions were met, results were interpreted directly.

#### 2.6.4. Segmented Regression Analysis

The relationship between watermeal supplementation level and PI was evaluated using segmented (broken-line) regression based on replicate-level data from T1–T4. Because PI is a composite production index and the fixed-diet experiment included only four supplementation levels, segmented regression was used to estimate a practically relevant breakpoint range under the present conditions rather than a precise biological optimum. The breakpoint and its 95% CI were estimated iteratively. Model fit was assessed using adjusted R^2^ and overall model significance. The model is expressed as:

*γ* = *γ*_0_ + *γ*_1_ × *SL* + *γ*_2_ × (*SL* > *xc*) × (*SL* − *xc*),

where *γ* represents the PI, *SL* is the supplementation level of watermeal (%), *γ*_0_ is the intercept, *γ*_1_ is the slope before the breakpoint (knot), and *γ*_2_ represents the change in slope after the breakpoint relative to the initial slope. The parameter xc denotes the estimated breakpoint. The indicator function (*SL* > *xc*) takes the value 1 when *SL* exceeds *xc* and 0 otherwise. Therefore, the term *γ*_2_ × (*SL* − *xc*) is included only when the supplementation level is greater than the breakpoint.

Diagnostic checks: Model adequacy was assessed based on residual plots, normality of residuals, and overall model fit (adjusted R^2^ and model significance). The stability of the breakpoint estimate was evaluated through CIs.

## 3. Results

### 3.1. Diet Composition

The ingredient composition and analyzed nutrient contents of the experimental diets are presented in Table 1. Increasing watermeal inclusion from 0% to 45% resulted in only minor variation in CP concentration (21.69–22.25%), suggesting that diets were approximately isonitrogenous. In contrast, CF and ash increased progressively with higher supplementation levels, reflecting the compositional characteristics of watermeal. GE ranged from 3917.52 to 3941.00 kcal/kg, indicating that the diets were also largely isoenergetic. The slight reduction in GE:CP ratio across treatments suggests a progressive narrowing of the dietary energy-to-protein balance, which may be biologically relevant to intake behavior and performance. Insects are known to regulate macronutrient intake under the framework of nutritional geometry. However, the present study did not directly assess intake targets, digestibility, or the physiological pathways underlying these responses. Therefore, these dietary differences are interpreted here as contextual features of the experimental diets rather than direct mechanistic evidence.

### 3.2. Self-Selection Feeding Behavior (T5)

Age-specific intake patterns under the self-selection regime are shown in Table 2. Total ADFI increased markedly from week 1 to week 3 and declined slightly in week 4, consistent with growth-stage-dependent consumption patterns.

Watermeal intake was absent in week 1 but increased significantly in weeks 3 and 4 (*p* < 0.05). The absence of watermeal intake in week 1 likely reflects early-life feeding behavior rather than a single causal factor, and may be influenced by developmental stage, novelty of the feed ingredient, palatability, particle size, and handling characteristics. The proportion of watermeal intake relative to total intake increased progressively from 13.10% (week 2) to 35.53% (week 4), indicating a clear ontogenetic shift in dietary selection once intake commenced. Over the 4-week period, cumulative intake was 948.98 mg/cricket, of which 25.56% consisted of watermeal. These results are consistent with age-dependent dietary selection and possible nutrient regulation, although early-stage intake patterns should be interpreted cautiously.

### 3.3. Nutrient and Energy Intake Under Self-Selection

Total nutrient and energy intake of crickets under the self-selection feeding regime (T5) is presented in Table 3. Over the 4-week experimental period, cumulative crude CP intake averaged 185.79 ± 14.50 mg/cricket. NFE intake was substantially higher (433.81 ± 33.85 mg/cricket), resulting in an NFE:CP ratio of 2.34 ± 0.01. Crude fat and CF intakes were 23.14 ± 2.01 and 35.78 ± 3.11 mg/cricket, respectively. GE intake reached 3.32 ± 0.26 kcal/cricket, with a GE:CP ratio of 17.85 ± 0.04 kcal/g CP. The relatively stable NFE:CP and GE:CP ratios indicate that, despite variation in ingredient selection across weeks, crickets approached a relatively consistent macronutrient intake balance under self-selection conditions.

### 3.4. Repeated-Measures Analysis of Production Performance

**Figure 1:**
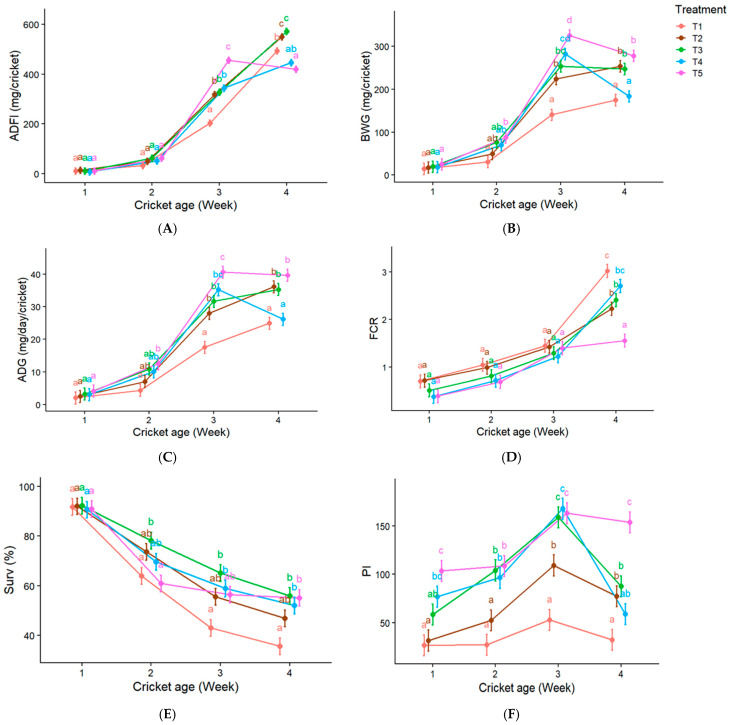
Repeated-measures analysis of production performance of crickets across weeks based on linear mixed-effects models (*n* = 30 experimental units). Panels show (**A**) Average daily feed intake; ADFI (mg/cricket), (**B**) Body weight gain; BWG (mg/cricket), (**C**) Average daily gain; ADG (mg/day/cricket), (**D**) Feed conversion ratio; FCR, (**E**) Survival rate; Surv (%), and (**F**) Production index; PI. Different lowercase letters (a–d) indicate significant differences among treatments (*p* < 0.05). Detailed statistical outputs, including effect sizes (partial R^2^) and confidence intervals, are provided in Supplementary Table S1.

Repeated-measures data were analyzed using linear mixed-effects models, with a replicate included as a random effect to account for within-unit dependence across time. The effects of treatment, week (age), and their interaction on production performance are summarized in Figure 1. Across all performance variables (ADFI, BWG, ADG, FCR, Surv, and PI), treatment, week, and their interaction were statistically significant (*p* ≤ 0.024). However, effect size analysis indicated that performance responses were primarily driven by the treatment × week interaction rather than by independent main effects (Supplementary Table S1).

Interaction effects were observed across all traits, indicating that differences among treatments emerged progressively over time. This pattern was the most pronounced for PI, suggesting that cumulative performance was particularly sensitive to time-dependent dietary responses.

Collectively, these results indicate that dietary effects on cricket performance were not static but developed across ontogeny. The significant interaction terms reflect non-parallel trajectories among treatments, with performance differences becoming more pronounced in later weeks, as illustrated in Figure 1.

### 3.5. Segmented Regression Analysis of PI

**Figure 2:**
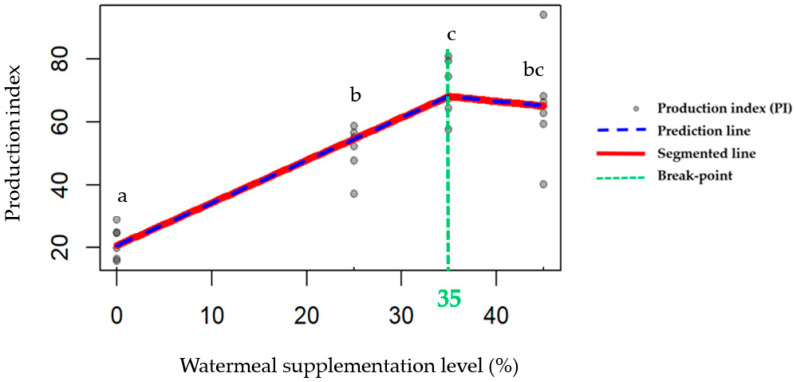
Segmented regression analysis of the PI during weeks 1–4 of the feeding trial, indicating a breakpoint at 35% watermeal supplementation (Adjusted R^2^ = 0.74, 95% confidence interval: 24.93–45.07; *p* < 0.001; *n* = 24). Different lowercase letters (a–c) above the PI symbol indicate statistically significant differences among treatments (*p* < 0.05).

The relationship between watermeal supplementation level and PI was evaluated using segmented regression analysis (Figure 2). Segmented regression analysis identified a breakpoint at 35% watermeal inclusion (95% CI: 24.93–45.07), indicating that, within the fixed-diet treatments evaluated, the highest PI occurred within a moderate supplementation range. The segmented model was highly significant (*p* < 0.001; *n* = 24) and explained a substantial proportion of the variance in PI (adjusted R^2^ = 0.74). The fitted equation was:

$$\gamma =20.76+\left(1.36\times SL\right)-1.67\times \left(SL>35\right)\times \left(SL-35\right),$$

where SL represents the level of watermeal supplementation (%).

The model indicates a positive linear increase in PI with increasing watermeal inclusion up to 35%, followed by a negative slope beyond this threshold. Specifically, PI increased by 1.36 units for each 1% increase in watermeal supplementation below 35%. In contrast, supplementation levels above 35% were associated with a decline in PI, with an estimated slope of −0.31 units per 1% increase (calculated as 1.36 − 1.67 = −0.31). These results indicate that performance was the highest within a moderate inclusion range centered near 35% under the present experimental conditions. However, given the discrete supplementation levels tested and the relatively broad 95% confidence interval of the breakpoint, this result should be interpreted as indicating a moderate performance-associated range rather than a precise single optimum.

### 3.6. Overall Growth Performance (Weeks 1–4)

Overall performance during the 4-week experimental period is presented in Table 4. Average daily gain increased significantly with watermeal inclusion, reaching the highest value in T5 (25.58 mg/day; *p* < 0.05). Body weight gain followed a similar pattern, with T5 achieving 715.77 mg per cricket. FCR improved progressively across treatments, with the lowest value observed in T5 (1.33), indicating enhanced feed efficiency. Surv was significantly lower in the control group (35.73%) compared with T3–T5 (≈52–56%). The PI was markedly higher in T5 (113.98), representing the greatest overall productivity among treatments. Because T5 was a self-selection treatment in which crickets chose between ingredients rather than consuming a fixed mixed diet, this result should be interpreted as reflecting performance under a distinct feeding architecture and not as direct evidence for a fixed formulated inclusion level.

### 3.7. Whole-Body Nutrient Composition

Whole-body proximate composition is presented in Table 5. CP content was significantly elevated in T2–T4 compared with T1 (*p* < 0.05), with T3 exhibiting the highest value (59.19%). Crude fat content decreased significantly in T3 and T4 relative to T1, indicating altered nutrient partitioning with moderate watermeal inclusion. GE content was significantly higher in T1 and T5 compared with intermediate treatments, likely reflecting higher lipid deposition in these groups. No significant differences were observed for CF, ash, calcium, or phosphorus.

## 4. Discussion

The present study demonstrates that the effects of watermeal supplementation on cricket performance are primarily developmentally mediated rather than driven by constant main effects. Although treatment and week were statistically significant in the linear mixed-effects models, their independent contributions to variance were negligible (partial R^2^ ≈ 0), whereas the treatment × week interaction consistently explained measurable variance across all performance traits (partial R^2^ = 0.043–0.092). This indicates that dietary effects emerged progressively over time and were strongly dependent on developmental stage. The results distinguish two complementary findings: (i) performance responses to defined dietary inclusion levels and (ii) behavioral regulation of nutrient intake under free-choice conditions. Segmented regression analysis within the fixed-diet treatments (T1–T4) identified an estimated breakpoint near 35% watermeal inclusion, indicating that moderate supplementation was associated with improved production performance under the present experimental conditions. Given the breadth of the confidence interval, this result is interpreted as a moderate performance-associated range rather than a precise single optimum.

### 4.1. Nutrient Balance and Dietary Geometry

Although diets were formulated to be approximately isonitrogenous and isoenergetic, increasing watermeal inclusion progressively altered CF and the dietary energy-to-protein balance, which may have contributed to differences in performance and body composition. In principle, the apparent discrepancy between protein content in the cricket body and the PI may be related to differences in intake pattern under self-selection, rather than by absolute protein intake alone. As shown in Table 3 and Table 4, the absolute CP intake of crickets was quantified for each treatment, with mean ± SD values of 140.96 ± 11.51, 181.84 ± 4.60, 192.10 ± 8.41, 169.12 ± 8.61, and 185.79 ± 14.49 mg CP per cricket over the experimental period for T1, T2, T3, T4, and T5, respectively. In treatments T1 and T4, crickets consumed relatively consistent proportions of CP due to fixed levels of watermeal supplementation. Across treatments T1 to T4, CP intake, ADG, and body CP content exhibited a generally linear relationship, indicating that CP deposition in the body was directly associated with protein intake and growth rate.

In contrast, PI did not consistently reflect CP deposition. This is expected because PI is a composite index integrating ADG, FCR, and Surv, and therefore reflects multidimensional performance outcomes rather than protein deposition alone. This explains the relatively high PI observed in T5 despite differences in body protein content.

In T5, where greater variability in intake occurred under self-selection conditions, the divergence between CP intake and body CP content may be interpreted within the framework of nutritional geometry [37,38]. Under this framework, animals regulate intake to approach a multidimensional nutrient target rather than prioritizing a single nutrient, such as protein. Accordingly, the observed intake pattern may reflect a balancing of nutrient sources. However, the present study did not directly measure digestibility, metabolism, or allocation of nutrients between growth and maintenance.

Even minor shifts in dietary macronutrient balance can influence feeding behavior and performance in insects, which are known to regulate intake toward specific protein–energy targets within the framework of nutritional geometry [39,40,41,42]. In orthopteran insects, performance is often maximized within a relatively narrow protein–carbohydrate intake range, and deviations from this intake target can reduce growth efficiency [30,41,43]. The segmented regression model used in the current study demonstrated a positive linear increase in PI up to 35% inclusion, followed by a decline beyond this breakpoint. The estimated breakpoint of 35% watermeal inclusion was associated with a relatively wide 95% CI (24.93–45.07%), indicating some uncertainty in the precise location of the breakpoint. Rather than representing a single fixed value, this interval suggests that performance was the highest across a biologically relevant moderate range of inclusion levels. This variability likely reflects both biological heterogeneity among replicates and the discrete supplementation levels evaluated in the present study. Importantly, this moderate range should be interpreted in the context of time-dependent responses, as the interaction-driven nature of the data indicates that the performance advantages of specific inclusion levels became evident primarily during later growth stages rather than uniformly across the entire production period. This range overlaps numerically with the voluntary intake observed under self-selection conditions (approximately 25–35%). However, the self-selection treatment differed fundamentally from the fixed-diet treatments in feeding architecture, so this overlap should be interpreted cautiously as contextual consistency rather than direct validation of the regression-derived breakpoint. Such nonlinear responses are consistent with classical nutrient requirement models reported in poultry [44,45] and swine [46], including cricket production [47,48]. At moderate inclusion levels (≈25–35%), watermeal may have provided a dietary balance that was more compatible with performance under the present conditions. However, higher inclusion levels were associated with higher fiber and ash contents, which may have contributed to lower performance. However, nutrient assimilation and digestibility were not directly measured in this study. In addition to macronutrient balance, these responses could also have been influenced by differences in nutrient digestibility, amino acid composition, and the presence of structural or anti-nutritional components associated with watermeal. For instance, increased dietary fiber could influence digestive processes or nutrient availability. In insects, dietary fiber and plant secondary compounds have been shown to modulate digestive processes and gut microbiota composition, which in turn can influence nutrient assimilation and growth performance [49,50]. However, the present study did not measure these factors directly and should therefore be interpreted with caution. These findings suggest that future feed formulation strategies should move beyond fixed inclusion levels toward nutrient-target-based design, where diets are formulated to achieve appropriate GE:CP ratios and digestible nutrient balance rather than relying solely on ingredient substitution percentages. From a formulation perspective, the results further indicate that watermeal may function not only as a replacement ingredient but also as a component within a balanced nutrient system.

Its practical inclusion may require consideration of fiber content, mineral load, and possible digestibility constraints, particularly at higher inclusion levels (>35%), where performance declined. Accordingly, future feed design may consider integrating (i) digestible nutrient fractions rather than total CP, (ii) fiber thresholds influencing intake and nutrient assimilation, and (iii) more direct indicators of energy–protein utilization.

### 4.2. Evidence of Nutritional Self-Regulation

Under self-selection conditions, crickets adjusted watermeal intake according to developmental stage. The progressive increase in watermeal consumption from week 2 onward (Table 2) and the stabilization of NFE:CP and GE:CP intake ratios (Table 3) are consistent with macronutrient regulation under choice conditions. However, the absence of watermeal intake in week 1 should be interpreted with caution. Early-stage refusal of watermeal may reflect multiple factors, including developmental stage, novelty of the feed ingredient, palatability, particle size, or handling characteristics, rather than nutrient regulation alone. Similar intake regulation has been documented in locusts and other orthopterans, which balance protein and carbohydrate intake to optimize growth and survival [41,51]. Importantly, the self-selection group achieved the highest ADG and PI values across weeks (Table 4), suggesting that voluntary nutrient balancing may enhance growth efficiency beyond fixed-ratio formulations. This finding aligns with the nutritional geometry theory, which predicts improved performance when insects are allowed to approach their preferred intake balance [41,52]. Importantly, the superior performance observed in the self-selection treatment (T5) reflects the ability of crickets to dynamically regulate nutrient intake rather than the effect of a fixed dietary composition. Therefore, T5 should not be interpreted as evidence that a single fixed inclusion level would achieve equivalent performance.

### 4.3. Ontogenetic Effects and Treatment Interactions

Although week (age) was statistically significant, its independent contribution to variance was negligible when partitioned using partial R^2^ (Figure 1). Instead, ontogenetic effects were expressed through interaction with dietary treatment, indicating that the developmental stage modulated the response to watermeal inclusion rather than acting as a dominant independent driver. Developmental stage strongly influences metabolic rate, nutrient demand, and tissue deposition in insects [38,53]. The significant treatment × week interactions indicate that dietary effects varied across developmental stages. Performance differences became more pronounced during later growth phases (weeks 3–4), which may indicate changing nutrient requirements as crickets approach maturity [54,55,56]. These findings emphasize the importance of stage-specific nutritional strategies and appropriate phase-specific feeding management in mass-reared insects [47]. The age-dependent changes in intake and performance observed in this study also suggest the existence of a dynamic nutritional profile across developmental stages. Early-stage crickets (weeks 1–2) exhibited minimal watermeal intake and lower total consumption, whereas later stages (weeks 3–4) showed increased intake and higher proportional inclusion of watermeal. This pattern may reflect a combination of developmental progression and gradual acceptance of the feed ingredient, rather than a direct response to nutrient requirements alone. Future studies should explicitly construct stage-specific nutritional profiles (e.g., protein:energy requirements across instars), which could support phase-feeding strategies analogous to those used in poultry and swine production.

### 4.4. Feed Conversion Efficiency and Nutrient Partitioning

FCR improved progressively with moderate watermeal supplementation and was lowest in the self-selection treatment. Improved FCR may reflect differences in nutrient utilization rather than simple increases in CP concentration, as diets were formulated to similar CP levels. The interaction-driven response observed in the present study suggests that feed efficiency responses were influenced by the interaction between dietary composition and developmental stage.

Whole-body composition data indicate that moderate watermeal inclusion (T2–T4) increased CP content while reducing crude fat deposition relative to the control diet. This shift in body composition is consistent with differences in nutrient use associated with dietary composition. In insects, carbohydrates are the primary substrates for glycolysis, generating ATP to support maintenance metabolism, locomotion, and anabolic processes [57,58,59]. Glucose derived from dietary NFE enters glycolysis and the TCA cycle, yielding ATP and metabolic intermediates required for biosynthesis. When carbohydrate intake exceeds immediate energy demands, excess acetyl-CoA derived from glycolysis can be redirected toward de novo lipid synthesis in the fat body [59,60,61]. Thus, the balance between carbohydrate supply and protein availability plays a central role in determining whether nutrients are allocated toward growth (protein accretion) or energy storage (lipid deposition) [62]. These pathways provide a conceptual framework for interpretation, but they were not directly measured in the present study.

In the present study, moderate watermeal inclusion slightly reduced the dietary GE:CP ratio while maintaining similar CP levels. This adjustment may have been associated with a more favorable dietary balance, but this interpretation should be considered hypothetical, as metabolic fluxes were not directly quantified. Adequate ATP availability is generally considered to be important for peptide bond formation, amino acid incorporation, and protein accretion, although these processes were not directly measured in the present study. However, when non-protein energy supply is excessive relative to protein, surplus carbohydrates may be diverted into lipid biosynthesis rather than supporting lean tissue growth. The reduced crude fat content observed in T3 and T4 may be consistent with altered nutrient allocation, although this mechanism was not directly assessed. Conversely, if dietary energy is insufficient relative to protein, amino acids may be deaminated and oxidized to meet energy requirements, reducing net protein retention [63,64]. The segmented regression analysis indicating an apparent optimum at approximately 35% watermeal supplementation may therefore reflect a dietary condition associated with improved overall production performance. However, this should be interpreted as a conceptual explanation rather than direct evidence of metabolic regulation.

The self-selection group (T5) maintained a relatively stable GE:CP intake ratio, indicating a consistent intake pattern under choice conditions. Such nutrient balancing is consistent with the framework of nutritional geometry, in which insects adjust feeding behavior to reach an intake target that maximizes growth and metabolic efficiency [57]. This pattern may be compatible with improved PI under balanced dietary conditions, although the underlying physiological mechanisms were not measured directly.

Collectively, these findings highlight the importance of aligning carbohydrate-derived energy supply with protein availability, which may be associated with differences in body composition and production performance in mass-reared orthopteran insects. It should be noted that ATP production, metabolic flux, and nutrient partitioning pathways were not directly measured in this study. Therefore, these mechanistic interpretations remain hypothetical and are intended to provide a physiological context for the observed performance responses. Furthermore, variation in amino acid balance and nutrient digestibility among treatments may also have contributed to differences in protein deposition efficiency, although amino acid profiles and digestibility coefficients were not determined in this study. Previous studies concerning insects and monogastric animals have demonstrated that imbalances in essential amino acids or reduced digestibility can limit protein accretion, even when CP levels are similar [65,66].

### 4.5. Voluntary Watermeal Intake Under Self-Selection

The self-selection treatment provided insight into the voluntary intake behavior of crickets under choice conditions. Over the 4-week experimental period, crickets in the self-selection group consumed watermeal at an average proportion of 25.56% of total intake, and this proportion increased progressively with age, reaching 35.53% at week 4 (Table 2). Importantly, the self-selection treatment addressed a different question from the fixed-diet experiment. Whereas the segmented regression analysis evaluated how PI responded to predefined watermeal inclusion levels in mixed diets (T1–T4), the self-selection treatment evaluated the intake proportion of crickets approached voluntarily when given a choice between ingredients. These two questions are related but not interchangeable, because T5 represented a distinct feeding architecture rather than a fixed formulated diet. The voluntary intake proportions observed in T5 were numerically similar to the moderate range identified in the fixed-diet analysis, but this overlap should not be interpreted as direct validation of the regression-derived breakpoint. Rather, the self-selection results are informative as behavioral evidence that crickets actively regulated ingredient intake over time and approached a moderate watermeal proportion under choice conditions.

The progressive increase in watermeal selection from week 2 onward further suggests the developmental modulation of nutrient demand. Early-stage crickets (week 1) did not consume watermeal, whereas older crickets incorporated it at increasing levels. This absence of intake in week 1 may reflect initial avoidance behavior or limited acceptance of the novel feed component, influenced by factors such as palatability, texture, particle size, or feeding experience, rather than nutrient requirements alone. As feeding progressed, increased intake likely reflects a combination of developmental changes and gradual adaptation to the available feed components.

From a practical perspective, the self-selection results suggest that moderate watermeal intake is behaviorally acceptable to crickets and may be compatible with good performance. However, these findings should be interpreted as complementary to, rather than confirmatory of, the fixed-diet performance analysis. Additional studies using more finely spaced fixed inclusion levels would be needed to define a more precise practical recommendation for formulated diets.

Taken together, the self-selection results provide behavioral evidence consistent with the statistically estimated optimal range identified in the dose–response analysis. These findings support a recommended watermeal supplementation range of approximately 30–35% in formulated diets for growing crickets under the present conditions. However, additional research across different rearing densities, genetic strains, and environmental conditions is required to further assess and confirm the robustness of this recommendation.

### 4.6. Practical Implications and Economic Considerations

The practical adoption of watermeal as a feed ingredient depends not only on biological performance but also on economic feasibility and production scalability. Although no direct cost–benefit analysis was conducted in this study, several indirect indicators suggest potential advantages. Watermeal can be cultivated rapidly in small-scale aquatic systems with minimal land requirements and high nutrient-use efficiency, particularly for nitrogen and phosphorus [20]. When integrated into circular production systems (e.g., wastewater or aquaculture effluents), it can simultaneously recover nutrients and generate biomass, potentially reducing production costs compared to conventional protein sources such as soybean meal [20]. In addition, its favorable protein profile and rapid renewability further support its potential as an alternative feed ingredient [67]. However, the economic viability of watermeal inclusion will depend on local production conditions, drying costs, and supply chain stability. Therefore, its application in commercial cricket production systems should be evaluated within region-specific techno-economic frameworks and validated through large-scale production studies [21].

### 4.7. Alternative Functional Feed Resources

Watermeal (*W. globosa*) shares functional characteristics with other emerging feed resources, including duckweed species (*Lemna* spp.), microalgae, and certain agricultural by-products with high protein content. These resources are similarly characterized by rapid biomass production, high nutritional value, and potential integration into circular production systems through nutrient recovery and reuse [68,69,70]. Comparative studies have shown that both duckweed and microalgae can effectively convert nutrients from waste streams into protein-rich biomass, although differences exist in growth dynamics, nutrient uptake, and processing requirements [71]. However, important knowledge gaps remain regarding digestibility, nutrient bioavailability, and practical feed utilization across these alternative resources [72]. Therefore, comparative evaluation of these feed sources in terms of digestibility, growth performance, and economic efficiency would provide valuable insights for optimizing feed system design in insect production.

### 4.8. Sustainability Framing

Watermeal (*W. globosa*) is a rapidly renewable aquatic biomass with characteristics compatible with circular agricultural systems, including high protein content, efficient nutrient uptake, and low land requirements [73,74,75]. However, it should be emphasized that the present study did not directly assess environmental impacts or sustainability metrics. Therefore, any claims regarding sustainability should be interpreted as conceptual or potential rather than empirically demonstrated within this experiment.

### 4.9. Study Limitations and Future Directions

Several limitations of the present study should be acknowledged. First, the experiment was conducted under controlled conditions using a single cricket strain and a fixed rearing density, which may limit the generalizability of the findings to other production systems. Second, although growth performance and body composition were evaluated, no direct measurements of digestibility, amino acid digestibility, digestive enzyme activity, metabolic biomarkers, calorimetric energy use, or gut microbiota were performed, limiting mechanistic interpretation. Third, environmental and economic impacts were not quantified, and thus the broader sustainability implications remain inferential.

Future research should consequently incorporate integrated approaches, including direct assessment of nutrient digestibility, amino acid composition, and potential anti-nutritional factors associated with watermeal. In addition, studies combining gut microbiota profiling, metabolomic analysis, and physiological indicators (e.g., digestive enzyme activity and metabolic biomarkers) would provide deeper insight into the mechanisms underlying watermeal-induced changes in growth performance and nutrient utilization. Such integrative approaches would be necessary to more directly test the mechanistic hypotheses raised by the present findings [76,77].

## 5. Conclusions

Dried watermeal (*W. globosa*) can be effectively incorporated into diets of two-spotted crickets (*G. bimaculatus*) at moderate inclusion levels, with segmented regression identifying an estimated breakpoint near 35% inclusion under the fixed-diet conditions tested. However, the associated confidence interval indicates that this result should be interpreted as a moderate performance-associated range rather than a precise single optimum. Performance followed a nonlinear and time-dependent dose–response pattern, with dietary effects expressed primarily through interaction with the developmental stage rather than through constant main effects. Under free-choice feeding, crickets actively regulated macronutrient intake and voluntarily approached moderate watermeal intake proportions over time, indicating behavioral intake regulation under choice conditions. Moderate supplementation improved feed efficiency and protein deposition while limiting lipid accumulation, which may be consistent with differences in nutrient balance under the present conditions. The integration of segmented regression and self-selection approaches provides a useful framework for evaluating performance responses and intake behavior in insect nutrition research and feed formulation studies. Collectively, these findings provide complementary statistical and behavioral evidence supporting a moderate inclusion range of approximately 30–35% watermeal in formulated diets. They also provide a basis for future studies using finer inclusion gradients and broader production conditions to refine practical recommendations.

## Figures and Tables

**Table 1:** Ingredient composition and the analyzed nutrient contents of the experimental diets used in the present study. Treatment 5 (T5) consisted of commercial feed (T1) and dried watermeal provided separately to the crickets.

Item	Dried Watermeal	Experimental Treatment			
		T1	T2	T3	T4
Ingredients					
Commercial diet (%)	0	100	75	65	55
Watermeal (%)	100	0	25	35	45
Analyzed nutrient composition					
Dry matter; DM (%)	92.26	87.75	88.88	89.33	89.78
Crude protein; CP(%)	22.94	21.69	22.00	22.13	22.25
Crude fat (%)	1.06	3.35	2.78	2.55	2.32
Crude fiber; CF (%)	6.82	3.31	4.19	4.54	4.89
Ash (%)	12.79	7.08	8.51	9.08	9.65
Nitrogen-free extract; NFE (%)	48.65	52.42	51.48	51.10	50.72
NFE:CP	2.12	2.42	2.34	2.31	2.28
Calcium (%)	0.62	1.05	0.94	0.90	0.86
Phosphorus, (%)	0.59	0.92	0.84	0.80	0.77
Gross energy; GE (kcal/kg)	3969.70	3917.52	3930.57	3935.78	3941.00
GE:CP (kcal:g CP)	17.30	18.06	17.86	17.79	17.71

**Table 2:** Feed intake (Mean ± SD) of crickets in treatment 5 (T5), representing the self-selection feeding group (*n* = 6).

Cricket Age (Week)	ADFI of Commercial Feed (mg/cricket)	ADFI of Watermeal (mg/cricket)	Total ADFI (mg/cricket)	Watermeal ADFI/Total ADFI (%)
1	10.13 ± 3.46 ^a^	0.00 ± 0.00 *	10.13 ± 3.46 ^a^	0.00 ± 0.00 *
2	55.61 ± 13.29 ^a^	9.69 ± 3.60 ^a^	62.07 ± 18.21 ^a^	13.10 ± 4.44 ^a^
3	371.24 ± 49.32 ^c^	85.23 ± 13.14 ^b^	456.47 ± 50.92 ^b^	18.79 ± 3.05 ^a^
4	268.37 ± 39.57 ^b^	151.94 ± 51.65 ^c^	420.31 ± 59.22 ^b^	35.53 ± 9.87 ^b^
1 to 4	706.38 ± 70.48	242.60 ± 46.03	948.98 ± 74.02	25.56 ± 4.46

^abc^ Different superscript letters within the same column of each cricket age indicate significant statistical differences (*p* < 0.05). * A value of zero was recorded for week-1 crickets. Therefore, this age group was not included in the statistical analysis. The absence of watermeal intake at this stage may reflect early developmental or behavioral factors (e.g., feed novelty, palatability, or physical characteristics of the diet) rather than any single causal factor. ADFI = Average daily feed intake.

**Table 3:** Calculated nutrient and gross energy (GE) intake (Mean ± SD) of crickets in treatment 5 (T5) under the self-selection feeding regime.

Item	Nutrient and Energy Intake						
	CP (mg/cricket)	Crude Fat (mg/cricket)	CF (mg/cricket)	NFE (mg/cricket)	NFE:CP	GE (kcal/cricket)	GE:CP (kcal:g CP)
Total intake	185.79 ± 14.50	23.14 ± 2.01	35.78 ± 3.11	433.81 ± 33.85	2.34 ± 0.01	3.32 ± 0.26	17.85 ± 0.04

CP = Crude protein, CF = Crude fiber, NFE = Nitrogen-free extract, and GE = Gross energy.

**Table 4:** Mean ± SD of growth performance and production index (PI) of crickets during the 4-week experimental period (*n* = 6).

Item	Experimental Treatment				
	T1	T2	T3	T4	T5
Average feed intake per cricket (mg/cricket)	740.60 ± 60.63 ^a^	929.96 ± 24.33 ^bc^	971.73 ± 42.91 ^c^	849.94 ± 43.65 ^b^	948.98 ± 74.02 ^c^
Body weight gain (mg)	360.39 ± 48.67 ^a^	544.02 ± 30.72 ^b^	596.45 ± 48.05 ^b^	553.93 ± 74.44 ^b^	715.77 ± 50.56 ^c^
Average daily gain (mg/day)	12.43 ± 1.68 ^a^	18.76 ± 1.06 ^b^	20.57 ± 1.66 ^b^	19.10 ± 2.57 ^b^	25.58 ± 2.66 ^c^
Feed conversion ratio (FCR)	2.07 ± 0.21 ^c^	1.71 ± 0.07 ^b^	1.64 ± 0.12 ^b^	1.55 ± 0.15 ^ab^	1.33 ± 0.14 ^a^
Survival rate (%)	35.73 ± 4.18 ^a^	46.96 ± 8.13 ^ab^	55.93 ± 3.92 ^b^	52.09 ± 8.85 ^b^	55.19 ± 13.82 ^b^
Production index	21.74 ± 5.22 ^a^	51.21 ± 7.92 ^b^	70.63 ± 9.10 ^c^	64.07 ± 3.92 ^c^	113.98 ± 18.40 ^d^
Sex ratio (male:female ratio)	1.08 ± 0.15	1.12 ± 0.21	1.02 ± 0.18	1.00 ± 0.12	1.06 ± 0.19

^abcd^ Different superscript letters within the same row indicate significant statistical differences (*p* < 0.05).

**Table 5:** Proximate nutrient and energy composition (Mean ± SD) of whole-body crickets (*n* = 6).

Item	Experimental Treatment				
	T1	T2	T3	T4	T5
Dry matter (%)	91.63 ± 0.13	91.34 ± 0.32	90.79 ± 0.51	90.66 ± 0.91	91.49 ± 0.61
Crude protein (%)	52.60 ± 0.87 ^a^	58.45 ± 2.03 ^bc^	59.19 ± 1.15 ^c^	57.96 ± 0.48 ^bc^	55.39 ± 2.39 ^ab^
Crude fat (%)	26.79 ± 0.64 ^c^	21.16 ± 2.14 ^ab^	19.59 ± 1.84 ^a^	19.82 ± 1.83 ^a^	24.10 ± 2.58 ^bc^
Crude fiber (%)	8.13 ± 0.83	7.18 ± 0.42	7.48 ± 0.20	7.87 ± 0.42	7.87 ± 0.86
Ash (%)	3.86 ± 0.26	4.45 ± 0.32	4.43 ± 0.36	4.53 ± 0.46	4.01 ± 0.48
Calcium (%)	0.32 ± 0.04	0.30 ± 0.06	0.29 ± 0.05	0.30 ± 0.04	0.27 ± 0.03
Phosphorus (%)	0.73 ± 0.06	0.80 ± 0.08	0.79 ± 0.14	0.75 ± 0.11	0.76 ± 0.10
Gross energy (kcal/kg)	5890.82 ± 15.33 ^b^	5658.95 ± 31.47 ^a^	5611.35 ± 28.20 ^a^	5578.05 ± 47.41 ^a^	5826.05 ± 67.56 ^b^

^abc^ Different superscript letters within the same row indicate significant statistical differences (*p* < 0.05).

## Data Availability

The original contributions presented in the study are included in the article/Supplementary Material, further inquiries can be directed to the corresponding author.

## Supplementary Materials

The following supporting information can be downloaded at: https://www.mdpi.com/article/10.3390/insects17040420/s1; Table S1: Effect size (partial R^2^) and 95% confidence intervals (CI) for fixed effects in linear mixed-effects models of production performance traits.

## Abbreviations

The following abbreviations are used in this manuscript:

ADFI	Average daily feed intake
ADG	Average daily gain
ANOVA	Analysis of Variance
ATP	Adenosine triphosphate
BWG	Body weight gain
CF	Crude fiber
CI	Confidence interval
CP	Crude protein
EBI	European Broiler Index
EPEF	European Production Efficiency Factor
FCR	Feed conversion ratio
GE	Gross energy
LCA	Life cycle assessment
LMM	Linear mixed-effects models
NFE	Nitrogen-free extract
PI	Production index
Q-Q	quantile–quantile
SD	Standard deviation
Surv	Survival rate
TCA	Tricarboxylic acid
