# Supplementary material for: Refining the Moderate Inclusion Range of Dried Asian Watermeal (Wolffia globosa) in the Diets of Two-Spotted Crickets (Gryllus bimaculatus): Integrating Segmented Regression and Nutritional Self-Selection
Source: Insects. 2026 Apr 15;17(4):420. doi: 10.3390/insects17040420 (PMC13116682; doi:10.3390/insects17040420)
Supplement: Supplementary file 1 [file insects-17-00420-s001.zip › insects-4195902-supplementary.pdf]

Table S1. Effect size (partial R^2) and 95% confidence intervals (CI) for fixed effects in linear mixed-effects models of production performance traits.

Item	Effect	Partial R^2*	95% Confidence interval	p-value
Average daily feed intake; ADFI (mg/cricket)	Treatment	0.000	0.00–0.05	<0.001
	Week	0.000	0.00–0.05	<0.001
	Treatment × Week	0.047	0.00–0.10	<0.001
Body weight gain; BWG (mg/cricket)	Treatment	0.000	0.00–0.11	<0.001
	Week	0.000	0.00–0.11	<0.001
	Treatment × Week	0.050	0.00–0.15	<0.001
Average daily gain; ADG (mg/day/cricket)	Treatment	0.000	0.00–0.10	<0.001
	Week	0.000	0.00–0.10	<0.001
	Treatment × Week	0.054	0.00–0.15	<0.001
Feed conversion ratio; FCR	Treatment	0.000	0.00–0.14	<0.001
	Week	0.000	0.00–0.14	<0.001
	Treatment × Week	0.063	0.00–0.19	<0.001
Survival rate; Surv (%)	Treatment	0.000	0.00–0.12	0.024
	Week	0.000	0.00–0.12	<0.001
	Treatment × Week	0.043	0.00–0.15	<0.001
Production index; PI	Treatment	0.000	0.00–0.17	<0.001
	Week	0.000	0.00–0.17	<0.001
	Treatment × Week	0.092	0.00–0.24	<0.001

* Partial R^2 values were calculated using the partR2 package to estimate the unique contribution of each fixed effect after accounting for shared variance. Confidence intervals were obtained via bootstrap resampling.
